# Mortalidade por Insuficiência Cardíaca e Desenvolvimento Socioeconômico no Brasil, 1980 a 2018

**DOI:** 10.36660/abc.20200902

**Published:** 2021-09-16

**Authors:** Sonia Carvalho Santos, Paolo Blanco Villela, Gláucia Maria Moraes de Oliveira

**Affiliations:** 1 Universidade Federal do Rio de Janeiro Rio de Janeiro RJ Brasil Universidade Federal do Rio de Janeiro – Cardiologia, Rio de Janeiro, RJ – Brasil

**Keywords:** Insuficiência Cardíaca, Indicadores de Desenvolvimento, Registros de Mortalidade

## Abstract

**Fundamento:**

Estudos sobre mortalidade por Insuficiência Cardíaca (IC) no Brasil e Regiões Geográficas (RG) são escassos.

**Objetivo:**

Analisar a evolução temporal das taxas de mortalidade por IC por sexo e faixa etária no Brasil, RG e Unidades da Federação (UF), de 1980 a 2018, e associações com o Índice de Desenvolvimento Humano Municipal (IDHM).

**Métodos:**

Estudo de séries temporais dos óbitos por IC, por sexo e faixas etárias, no Brasil, RG e UF, de 1980 a 2018. Os óbitos e a população foram retirados do DATASUS para estimar taxas de mortalidade por 100.000 habitantes, brutas e padronizadas (método direto, população brasileira do ano 2000). Foram calculadas médias móveis de três anos das taxas padronizadas. Os IDHM das UF de 1991 e 2010 foram obtidos do Atlas Brasil. Empregou-se o coeficiente de correlação de Pearson, com 5% de significância.

**Resultados:**

A mortalidade por IC diminuiu no Brasil a partir de 2008, atingindo ao final de 2018 patamar semelhante nas RG e UF, sendo maior nos homens durante quase todos os períodos e faixas etárias, exceto naqueles acima de 60 anos, a partir de 1995, na região Sul. Observou-se relação inversa entre o IDHM e a redução das taxas de mortalidade (0,73).

**Conclusão:**

Houve redução das taxas de mortalidade por IC no Brasil progressivamente de 2008 até 2018, com patamares semelhantes em 2018 nas RG e UF, com maiores taxas no sexo masculino. Essas reduções parecem relacionadas com o IDHM em 2010, mais do que o aumento percentual ao longo do tempo.

## Introdução

A mortalidade anual por Doenças Cardiovasculares (DCV) é maior do que por qualquer outra causa, fazendo desta a primeira causa de óbitos no mundo. Estima-se que 17,9 milhões de pessoas morreram de DCV em 2016, representando 31% de todas as mortes globais e mais de três quartos das mortes por DCV ocorrem em países de baixa e média renda.^1^ Dentre as DCV a insuficiência cardíaca (IC) destaca-se pela elevada e crescente morbidade e mortalidade.^2^

Dados do *Global Burden of Disease (* GBD) não disponibilizam estimativas de mortalidade por IC por considerá-la a via final comum de várias doenças, caracterizando-a como *garbage code* (i.e, um código inespecífico, incompleto e que não identifica claramente a causa básica do óbito),^3^ e redistribuindo as mortes pelas condições que foram responsáveis por sua ocorrência. No Brasil, segundo o GBD, a prevalência e a taxa padronizada por IC, por100mil habitantes, em 1990 e 2017, foi de 670.194,8 (II_95_=589952,6; 753.672,6) e 818,1 (II_95=_718,1; 922,8), 1.686.320,1 (II_95=_1.478.563,8;1.890.537,3) e 777,2 (II_95=_ 680,0;874,80), respectivamente, com redução percentual de -5% (II_95-_ -7.1;-3) na taxa de prevalência padronizada ao longo de 27 anos.^3^

Os estudos sobre mortalidade por IC no Brasil, com dados do Sistema de Informação de Mortalidade em Regiões Geográficas (RG) e Unidades da Federação (UF) com diferentes níveis de desenvolvimento socioeconômico são escassos. Estudo realizado entre os anos de 2004 e 2011, considerando-se as causas básicas de morte, observou que a mortalidade proporcional por IC aumentou com a idade, e as maiores porcentagens foram notadas entre as mulheres idosas, no Brasil e RG.^4^ Os autores fizeram associação com a doença isquêmica do coração como causa mais frequente para a ocorrência e desenvolvimento de IC, e discutiram as diferenças regionais como consequência, entre outras, das condições socioeconômicas e estruturas de atenção à saúde.^5^ Recente estudo realizado no estado da Paraíba, estado com menor desenvolvimento socioeconômico, entre 2008 e 2015, reportou que a mortalidade por IC em números absolutos apresentou um declínio não significativo de 2008 a 2015 (R = -0,513), o mesmo acontecendo no Brasil (R = -0,412), sem diferença estatisticamente significativa quanto ao gênero e faixas etárias.^6^ No entanto, as relações da IC com os indicadores sociais e econômicos no Brasil foram pouco exploradas na literatura até o momento.

O Índice de Desenvolvimento Humano (IDH), que pretende representar saúde, educação e renda, na tentativa de medir vida longa e saudável, acesso ao conhecimento e padrão de vida, parece ser um bom indicador socioeconômico para avaliação dessas relações complexas entre os determinantes sociais e DCV. Desde 2009, é composto pela expectativa de vida ao nascer, por anos médios de estudo da população adulta e anos esperados de estudo para as crianças (taxa de matrícula escolar), e pela renda *per capita.* ^7^

Esse estudo pretende analisar a evolução temporal das taxas de mortalidade por IC de acordo com sexo e faixa etária no Brasil, nas RG e nas UF ao longo dos últimos 39 anos, e as associações com o IDH, índice escolhido para comparar o desenvolvimento socioeconômico entre as UF.

## Materiais e Métodos

Trata-se de estudo ecológico e descritivo de séries históricas de registro de óbitos por Insuficiência Cardíaca (IC) ocorridos no Brasil, entre 1980 e 2018, em todas as faixas etárias e em ambos os sexos.

As informações sobre a causa básica de óbito foram retiradas do *site* do Sistema de Informações sobre Mortalidade (SIM) do Departamento de Informática do Sistema Único de Saúde (DATASUS) do Ministério da Saúde.^8^ Após o *download* da base dados, os arquivos originais em formato CSV foram convertidos em formato XLS através do programa Microsoft Excel,^9^ o mesmo utilizado para análise de dados e construção de gráficos e tabelas. Para identificar os óbitos cuja causa básica foram IC, utilizou-se as categorias 428 da CID-9^10^ para os óbitos ocorridos entre 1980 e 1995, e I50 da CID-10^11^ para os óbitos ocorridos a partir de 1996.

As informações sobre a população residente foram também retiradas do *site* do DATASUS,^8^ que por sua vez considerou os dados censitários do Instituto Brasileiro de Geografia e Estatística (IBGE) de 1980, 1991, 2000 e 2010, projeções intercensitárias até 2012, e projeções populacionais de 2013 em diante.

Foram estimadas as taxas de mortalidade anuais, nas UF, por 100.000 habitantes, brutas e padronizadas pelo método direto,^12^ utilizando-se como padrão a estrutura etária da população brasileira do ano 2000. Para cada UF foram calculadas as médias móveis das taxas padronizadas a cada três anos, desconsiderando-se os dois anos iniciais da série (1980 e 1981 para todas as UF; 1989 e 1990 para Tocantins), até 2018. As UF foram agrupadas nas cinco RG do país (Norte, Nordeste, Sudeste, Sul e Centro-Oeste). Ressalta-se que a partir de 1989, a região Norte passou a computar os dados de Tocantins, UF criada em 1988.

Foram estimadas as taxas brutas de mortalidade por região geográfica, em três faixas etárias (até 29 anos, 30-59 anos, 60 anos ou mais), em sete períodos de cinco anos e em um período de quatro anos (2015 a 2018), com posterior cálculo da razão das taxas para os sexos masculino/feminino.

O Índice de Desenvolvimento Humano (IDH) de cada UF correspondente aos anos de 1991 e 2010 foram obtidos do *site* Atlas Brasil.^13^ As informações são resultado da adaptação do cálculo do IDH global do país para os níveis municipal e estadual, realizados pelo Programa das Nações Unidas para o Desenvolvimento (PNUD - Brasil), pelo Instituto de Pesquisa Econômica Aplicada (IPEA) e pela Fundação João Pinheiro, criando assim o Índice de Desenvolvimento Humano Municipal (IDHM), cuja interpretação é a mesma do IDH global, porém em níveis municipal e estadual. A seguir, calculou-se a variação percentual do IDHM de cada UF entre 1991 e 2010, e a sua correlação com a variação percentual das taxas de mortalidade padronizadas nas respectivas UF entre 1990 e 2018 empregando-se o coeficiente de correlação de Pearson, para o qual foi adotado nível de significância inferior a 0,05. Ressalta-se que, neste caso, foi escolhido o ano de 1990 para início da série temporal para que todas as UF pudessem ser avaliadas com o mesmo intervalo de tempo, considerando a criação de Tocantins em 1988. Procedeu-se também a realização da correlação do IDHM de 2010 com a variação percentual das taxas de mortalidade padronizadas nas respectivas UF entre 1990 e 2018, dado disponibilizado com a atual metodologia de cálculo.

## Resultados

Entre 1980 e 2018 foram encontrados 1.185.120 óbitos, sendo 49,3% (584.155) no sexo masculino. Quanto a distribuição por RG, 48.533 ocorreram na Região Norte, 245.898 na Região Nordeste, 602.105 na Região Sudeste, 218.496 na Região Sul e 70.088 na Região Centro-Oeste. Os dados completos empregados para o estudo estão disponibilizados nos anexos 1 , 2 , 3 e 4 .

**Figura 1:**
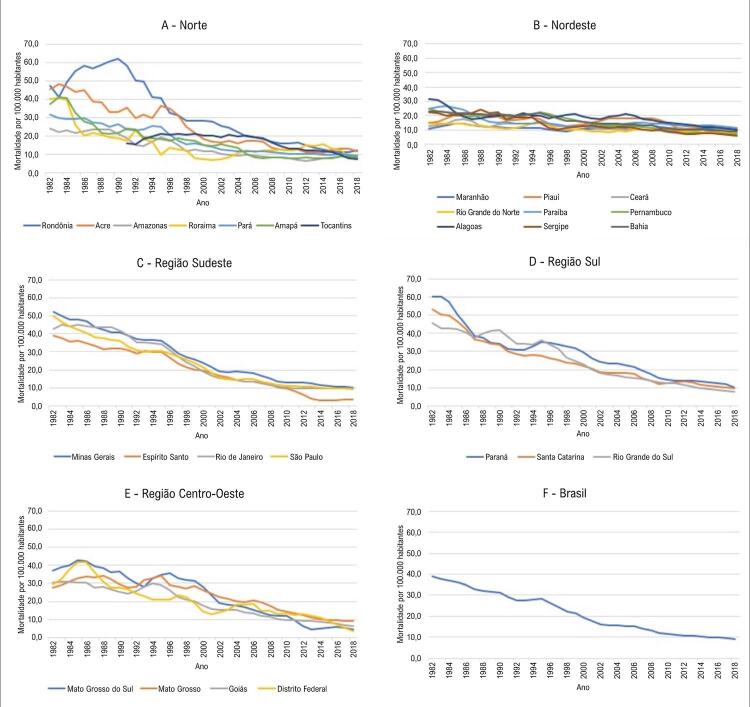
– *Médias móveis de três anos das taxas de mortalidade padronizadas por idade, por 100.000 habitantes, em cada Unidade da Federação agrupadas nas cinco Regiões Geográficas (Figuras 1A- Norte, IB- Nordeste, IC- Sudeste, ID Sul, IE- Centro-Oeste) e o total nacional (1F).*

A Figura 1 apresenta as médias móveis de três anos das taxas de mortalidade padronizadas por idade, por 100.000 habitantes, em cada UF agrupadas nas cinco regiões geográficas ( Figuras 1A a 1E ) e o total nacional ( Figura 1F ), no período entre 1982 e 2018. Na região Norte, à exceção de Rondônia e Acre, que apresentaram aumento das médias na primeira e segunda décadas de observação, respectivamente, todas as demais UF apresentaram declínio progressivo e, a partir de 2008, as médias foram semelhantes em todas as UF com pequenas oscilações até 2018 ( Figura 1A ). Tocantins, por ter sido criado em 1988, apresentou dados a partir de 1989 e neste caso, o início do cálculo das médias móveis ocorreu a partir de 1991 ( Figura 1A ). Na região Nordeste ( Figura 1B ), Alagoas apresentou as maiores médias no início do período e, apesar da tendência de declínio, mostrou elevações entre 1998 e 2008, comportamento semelhante ao do Piauí. Seguindo a mesma tendência da Região Norte, a partir de 2008, as médias de todas as UF da Região Nordeste foram semelhantes entre si, apresentando a mesma tendência evolutiva nos último 10 anos de observação.

As UF da região Sudeste ( Figura 1C ), apesar de mostrarem médias elevadas no início do período, apresentaram queda progressiva ao longo dos anos, em especial no Espírito Santo, onde a partir de 2010 se destacou por apresentar as médias mais baixas da região, em caráter estável e sustentado. As UF da região Sul ( Figura 1D ), assim como observado na região Sudeste, apresentaram médias elevadas no início do período de observação e, à exceção do Paraná que apresentou elevação durante toda a década de 90, todas as UF apresentaram queda progressiva, atingindo valores semelhantes aos da região Sudeste no período final de observação. A Figura 1E mostra importantes oscilações nas UF da região Centro-Oeste ao longo das três primeiras décadas, assumindo tendência a linearidade apenas nos últimos 10 anos de observação. Assim como visto nas regiões em separado, a tendência nacional no período ( Figura 1F ) é de queda. Partindo-se de valores intermediários no início da série, ocorreram pequenas oscilações, sobretudo na década de 1990, com posterior tendência a linearidade a partir do início dos anos 2000.

A Tabela 1 apresenta a razão das taxas de mortalidade entre os sexos masculino e feminino nas cinco regiões geográficas, em períodos de cinco anos, em três faixas etárias. As taxas no sexo masculino foram maiores durante quase todos os períodos e faixas etárias observadas, atingindo as maiores proporções na faixa etária entre 30-59 anos em todas as regiões geográficas. As taxas de mortalidade no sexo feminino foram superiores (razão <1) apenas na faixa etária até 29 anos em breves períodos nas regiões Norte e Nordeste, e na faixa etária acima de 60 anos, a partir de 1995, na região Sul ( Tabela 1 ).

**Tabela 1:** – Razão entre as taxas bruta de mortalidade nos sexos masculino e feminino, e grupos etários, em períodos de cinco anos, por região geográfica

Faixa etária	Região/Período	1980-1984	1985-1989	1990-1994	1995-1999	2000-2004	2005-2009	2010-2014	2015-2018
0-29	Norte	1,0	0,9	1,2	1,0	1,2	1,3	1,3	1,8
	Nordeste	0,9	1,0	1,0	1,1	1,1	1,4	1,4	1,5
	Sudeste	1,1	1,2	1,3	1,3	1,2	1,6	1,5	1,8
	Sul	1,1	1,2	1,2	1,6	1,5	1,3	1,5	1,1
	Centro-Oeste	1,1	1,1	1,1	1,1	1,9	1,5	2,7	1,0
30-59	Norte	1,5	1,4	1,5	1,4	1,6	1,9	1,8	1,5
	Nordeste	1,2	1,3	1,4	1,3	1,3	1,4	1,5	1,6
	Sudeste	1,4	1,5	1,6	1,5	1,6	1,7	1,6	1,5
	Sul	1,4	1,5	1,5	1,4	1,4	1,5	1,4	1,2
	Centro-Oeste	1,2	1,5	1,6	1,6	1,8	1,9	1,7	1,7
60+	Norte	1,1	1,1	1,1	1,1	1,2	1,3	1,3	1,2
	Nordeste	1,2	1,2	1,2	1,1	1,2	1,2	1,2	1,2
	Sudeste	1,1	1,1	1,0	1,0	1,0	1,0	1,0	1,0
	Sul	1,1	1,0	1,0	0,9	0,9	0,9	0,9	0,9
	Centro-Oeste	1,1	1,1	1,0	1,0	1,1	1,1	1,1	1,2

**Figura 2:**
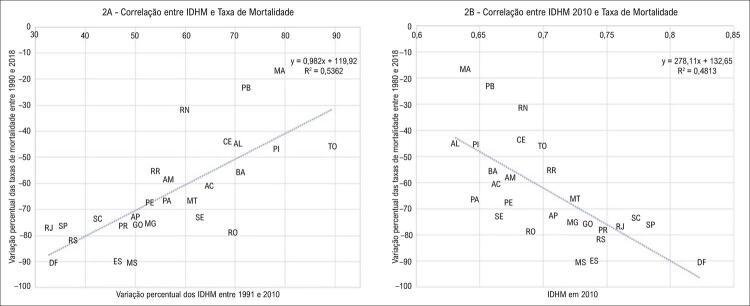
– *Gráficos de dispersão. A) Correlação entre as variações percentuais do IDHM entre 1991 e 2010 e as taxas de mortalidade entre 1990 e 2018, em cada Unidade da Federação (Siglas) do Brasil. B) Correlação entre o IDHM absoluto no ano 2010 e a variação percentual das taxas de mortalidade entre 1990 a 2018, em cada Unidade da Federação (siglas) do Brasil.*

O coeficiente de correlação de Pearson entre a variação das taxas de mortalidade entre 1990 e 2018 e a variação do IDHM entre 1991 e 2010 de cada UF foi 0,73 (correlação forte) com p=0,00001, e a Figura 2A apresenta as UF em gráfico de dispersão, enquanto a Figura 2B demonstra a correção das taxas de mortalidade e o IDHM de 2010, com valor de 0,72. Como já observado na Figura 1 em relação as médias móveis em período mais prolongado, todas as UF apresentaram redução e, portanto, variação negativa nas taxas de mortalidade, quando comparados os anos de 1990 e 2018 ( Figura 2A , eixo y). Por outro lado, todas as UF apresentaram aumento e, portanto, variação positiva nos IDHM entre 1991 e 2010 ( Figura 2A , eixo x). Pode-se notar na Figura 2A , as UF que apresentaram as maiores reduções nas taxas de mortalidade foram as que apresentaram os menores aumentos no IDHM. Ao contrário, as UF que apresentaram as menores reduções nas taxas de mortalidade foram as que apresentaram os maiores aumentos no IDHM. A Figura 2B demonstra a relação inversa entre o IDHM 2010 e as variações percentuais das taxas de mortalidade. A Tabela 2 apresenta os IDHM de 2010, e as variações do IDHM entre 1991 e 2010 de cada UF.

**Tabela 2:** – Índice de Desenvolvimento Humano Municipal (IDHM) por Unidade da Federação e sua variação percentual entre 1991 e 2010

Unidade da Federação	IDHM 2010	∆% 1991-2010
Rondônia	0,690	69,5
Acre	0,663	64,9
Amazonas	0,674	56,7
Roraima	0,707	54,0
Pará	0,646	56,4
Amapá	0,708	50,0
Tocantins	0,699	89,4
Maranhão	0,639	79,0
Piauí	0,646	78,5
Ceará	0,682	68,4
Rio Grande do Norte	0,684	59,8
Paraíba	0,658	72,3
Pernambuco	0,673	53,0
Alagoas	0,631	70,5
Sergipe	0,665	63,0
Bahia	0,660	71,0
Minas Gerais	0,731	52,9
Espírito Santo	0,740	46,5
Rio de Janeiro	0,761	32,8
São Paulo	0,783	35,5
Paraná	0,749	47,7
Santa Catarina	0,774	42,5
Rio Grande do Sul	0,746	37,6
Mato Grosso do Sul	0,729	49,4
Mato Grosso	0,725	61,5
Goiás	0,735	50,9
Distrito Federal	0,824	33,8

*∆% 1991-2010 = Variação percentual entre 1991 e 2010*

## Discussão

A IC afeta aproximadamente 26 milhões de pessoas em todo o mundo, esses dados tendem a aumentar com o envelhecimento populacional, com a alta prevalência de fatores de risco cardiovascular, com a sobrevivência dos pacientes a eventos coronarianos agudos e com melhorias terapêuticas da IC.^14^ Nos Estados Unidos da América, estima-se que até 2030, mais de 8 milhões de pessoas terão a doença, com números crescentes devido ao envelhecimento populacional.^15^

A mortalidade por IC diminuiu no Brasil ao longo dos 29 anos estudados, apresentando tendência de redução progressiva a partir de 2008, atingindo ao final de 2018 patamar semelhante nas RG e UF ( Figura 1 ). Essa tendência foi semelhante a observada em estudo com 5.823 pacientes seguidos por um ano em diferentes regiões do mundo, e que apontou mortalidade proporcional de 9% na América do Sul. Os autores observaram mortalidade elevada na África (34%) e na Índia (23%), intermediária no Sudeste Asiático (15%), e menor na China e no Oriente Médio (7%), que persistiram apesar do ajuste por múltiplas variáveis clínicas, terapêutica medicamentosa, e fatores socioeconômicos. Os autores formularam a hipótese de que a qualidade, o acesso, e a infraestrutura dos serviços de saúde, bem como fatores genéticos e ambientais estariam envolvidos nesse complexo fenômeno.^16^

Outro aspecto importante é que a idade média dos pacientes com IC era uma década menor nos países de baixa-média renda quando comparado com os de alta renda,^17^ o que pode estar relacionado com o retardo no diagnóstico e tratamento, que acarretaria pior prognóstico para os pacientes menos favorecidos e que se somaria a baixa expectativa de vida nesses países.^14 , 18^ Em coorte de 4 milhões de indivíduos representativa da população do Reino Unido, oriunda da *Clinical Practice Research Datalink (CPRD),* observou-se que indivíduos desfavorecidos do ponto de vista socioeconômico foram mais propensos a desenvolver IC do que os indivíduos ricos (razão de taxa de incidência 1:61, IC_95%_ 1,58-1,64), e o fizeram mais cedo na vida (diferença ajustada –3,51 anos, IC_95%_ 3,77–3,25), com mais comorbidades, apesar de mais jovens. Também notaram que de 2002 a 2014, o gradiente socioeconômico da idade na primeira apresentação com IC aumentou.^19^

As taxas de mortalidade por IC no sexo masculino foram maiores durante quase todos os períodos e faixas etárias observadas, exceto na faixa etária acima de 60 anos, a partir de 1995, na região Sul ( Tabela 1 ), provavelmente relacionada com a etiologia isquêmica da IC, exceto nas idades mais avançadas, o que pode estar associado com a maior longevidade das mulheres, conforme observado em metanálise que reuniu cerca de 240 mil pacientes com IC aguda e crônica.^17^ Outro estudo com 88.416 pacientes com a base de dados *Clinical Practice Research Datalink (CPRD)* do Reino Unido, observou que os riscos de desfechos adversos foram maiores nos mais velhos, nos homens, nos com privação socioeconômica e naqueles cujo diagnóstico de IC foi realizado quando da hospitalização. Notaram também piora dos desfechos em mulheres nas últimas duas décadas. Os autores concluíram que essas disparidades provavelmente refletem a carga crescente de doenças não cardiovasculares em pacientes com IC, que exigirão mudança da abordagem contemporânea, que também precisará agregar a gestão e melhoria do status socioeconômico.^20^

Estudos prévios mostraram que em países onde o IDH é baixo, os pacientes apresentam IC em idade mais jovem do que nos países com IDH mais elevado,^17^ e a privação econômica está associada com maior incidência de IC em nível nacional.^21 , 22^ Em estudo com mais de 17.100 pacientes de um Sistema Universal de Saúde, com IC e fração de ejeção do ventrículo esquerdo reduzida, observou-se que a renda baixa foi associada com maior risco de morte por todas as causas, readmissão nos 12 meses subsequentes ao diagnóstico de IC, maior tempo de internação, e maior taxa de mortalidade hospitalar.^23^

Observou-se tendência inversa entre a variação da taxa de mortalidade das UF entre 1990 e 2018, e a variação do respectivo IDHM entre 1991 e 2010. Assim, embora as UF que apresentaram as maiores reduções nas taxas de mortalidade tenham apresentado os menores incrementos no IDHM ( Figura 2A - RJ, DF, SP, RS, SC, ES), todas atingiram IDHM igual ou superior a 0,7 em 2010 ( Tabela 2 ). Ao contrário, observou-se que nenhuma das UF com os maiores incrementos no IDHM ( Figura 2A - TO, MA, PI, PB, AL, BA) apresentou IDHM maior que 0,7 em 2010 ( Figura 2B ). Este fato sugere que em relação a mortalidade por IC, mais importante que o grau de incremento do IDHM é o nível final que ele alcança. Estudo que avaliou 1802 pacientes do Reino Unido com IC e fração de ejeção do ventrículo esquerdo reduzida, empregando um Índice Múltiplo de Privação Socioeconômica, observou que mortalidade por todas as causas e a mortalidade por causas não cardíacas, ajustada pela idade, foram associadas com alto risco de privação socioeconômica, mas não com a mortalidade por causas cardiovasculares. Esse excesso de risco foi atribuído ao excesso de mortalidade não cardíaca e hospitalizações e não pode ser associado com a falta de medicação para IC baseada em evidências. Os autores sugerem que intervenções socioeconômicas precisam ser implementadas para reduzir os riscos pessoais e a carga econômica da doença em pacientes com IC e baixo nível de *status* socioeconômico.^24^

No presente estudo não foram avaliadas as causas múltiplas de óbito, somente as causas básicas selecionadas a partir das informações registradas nas declarações de óbito. Este fato torna-se uma limitação porque os códigos relacionados a IC, em geral, são descartados após a aplicação das regras de seleção de causa básica da Organização Mundial da Saúde,^25^ o que pode levar ao subdimensionamento dos óbitos por IC. Entretanto, por serem regras de aplicabilidade mundial, acredita-se que não haja prejuízo quando realizada a comparação de mortes entre diferentes países e/ou regiões.

Outro ponto a ser destacado é que por tratar-se de um estudo que avalia diretamente a causa básica de óbito, a qualidade desta informação depende do adequado preenchimento da declaração de óbito. Erros no preenchimento e incompletude das declarações ocasionados por desconhecimento do declarante^26^ representam potenciais problemas que podem interferir nas estatísticas oficiais. Entretanto, por ser de caráter sistêmico, possíveis erros afetariam toda as causas de óbito não influenciando apenas nos óbitos por IC.

O IDH por sua vez, apesar de incluir dados relacionados a renda, escolaridade e expectativa de vida, representa apenas uma visão parcial do *status* socioeconômico de determinado país ou região, não sendo possível a avalição de fenômenos como desigualdade ou qualidade de vida e suas influências na mortalidade por IC. Entretanto, por sua disponibilidade mundial permite comparar, com adequada dimensão, diferentes populações.

A IC representa enorme ônus econômico para a sociedade, sendo a principal causa de hospitalização nos países ocidentais.^27^ Nos países em desenvolvimento e com muitas desigualdades sociais, com prevalência crescente, especialmente nos mais jovens, e com gastos públicos ineficientes em assistência médica, os efeitos econômicos da IC a longo prazo precisarão ser considerados, principalmente em países continentais como o Brasil.

Poucos dados são conhecidos sobre a epidemiologia da IC, especialmente em países de média-renda como o Brasil, onde acredita-se que a prevalência está aumentando, e reporta-se associação com a doença isquêmica do coração, doença reumática, doença de Chagas e hipertensão, entre outras.^28^ A redução da mortalidade por IC pode ser consequência dos avanços no tratamento da Doença Isquêmica do Coração (DIC), mas também deve estar relacionada com a evolução do tratamento da própria IC, principalmente após a introdução do bloqueio neuro-humoral.^4^

^.^ Esforços devem ser feitos no sentido de ampliar o acesso à assistência à saúde e o controle mais efetivo dos fatores de risco cardiovasculares, dislipidemia, obesidade, sedentarismo, diabetes, bem como dos determinantes sociais, que contribuem tanto para a mortalidade por DIC quanto por IC. E é neste contexto que pode ter papel importante a ampliação da abrangência do Programa de Saúde da Família, que além de converter o modelo de cuidado para a atenção básica, aumenta a cobertura do Sistema Nacional de Saúde, reduzindo a proporção de mortes não assistidas, melhorando a qualidade da informação vital no Brasil, e diminuindo as hospitalizações por doenças crônicas como a IC.^29^ Estudos futuros precisarão ser realizados relacionando a capacidade instalada de recursos de saúde, e as causas múltiplas representadas pelos fatores de risco como contribuintes para o processo complexo da morte, a fim de que possamos direcionar as políticas de saúde pública voltadas para IC no Brasil.

## Conclusão

Este estudo avaliou a mortalidade por IC no Brasil ao longo de 39 anos, em cada UF das RG e demonstrou que, apesar de oscilações, todas as UF apresentaram redução das taxas de mortalidade especialmente nos últimos 10 anos de observação. Observou-se que nas faixas etárias entre 30-59 anos houve predomínio dos óbitos no sexo masculino. Houve tendência a relação inversa entre os percentuais de aumento do IDHM e redução das taxas de mortalidade, podendo esta última estar relacionada ao nível absoluto de IDHM alcançado em 2010. Estes achados poderiam, ao menos em parte, se justificar por melhorias no acesso ao sistema de saúde, no tratamento da IC, e nas condições socioeconômicas da população ao longo de quase quatro décadas.

## * Material suplementar

Para informação adicional, por favor, clique aqui.


