# Supplementary material for: Mortalidade por Insuficiência Cardíaca e Desenvolvimento Socioeconômico no Brasil, 1980 a 2018
Source: Arq Bras Cardiol. 2021 Sep 16;117(5):944–51. [Article in Portuguese] doi: 10.36660/abc.20200902 (PMC8682101; doi:10.36660/abc.20200902)
Supplement: Supplementary file 1 [file 2020-0902_material_suplementar.pdf]

Anexo I - População residente, por Unidade da Federação - Brasil, 1980-2018.

Unidade da Federação	1980	1981	1982	1983	1984	1985	1986	1987	1988
Rondônia	490405	540684	601105	661736	722207	782482	842192	901050	958688
Acre	300813	310305	321268	332269	343240	354176	365010	375689	386147
Amazonas	1428536	1482142	1545533	1609143	1672586	1735823	1798467	1860217	1920688
Roraima	79056	89863	102898	115979	129025	142029	154910	167608	180043
Pará	3399574	3522990	3668640	3814793	3960563	4105858	4249793	4391673	4530614
Amapá	175187	184083	194832	205617	216375	227097	237719	248190	258443
Tocantins									
Maranhão	3991595	4068616	4156556	4244801	4332814	4420540	4507445	4593109	4677000
Piauí	2137390	2173286	2215014	2256887	2298649	2340276	2381513	2422161	2461967
Ceará	5283123	5371642	5473194	5575098	5676734	5778039	5878395	5977319	6074194
Rio Grande do Norte	1896872	1938181	1986904	2035795	2084558	2133163	2181312	2228774	2275253
Paraíba	2766680	2803499	2844081	2884802	2925417	2965900	3006003	3045534	3084247
Pernambuco	6137758	6219407	6312125	6405164	6497958	6590451	6682077	6772395	6860843
Alagoas	1980299	2023691	2073743	2123968	2174062	2223992	2273455	2322212	2369959
Sergipe	1138999	1167321	1200446	1233685	1266837	1299881	1332616	1364883	1396483
Bahia	9440698	9640986	9868279	10096357	10323837	10550577	10775194	10996603	11213428
Minas Gerais	13370836	13561400	13784075	14007518	14230376	14452508	14672561	14889471	15101890
Espírito Santo	2021360	2067979	2122341	2176892	2231299	2285529	2339251	2392206	2444064
Rio de Janeiro	11276933	11408762	11551541	11694814	11837711	11980142	12121241	12260324	12396527
São Paulo	25025680	25547066	26163713	26782488	27399638	28014784	28624170	29224852	29813099
Paraná	7624132	7692748	7769903	7847325	7924544	8001512	8077759	8152917	8226519
Santa Catarina	3625071	3698615	3784692	3871066	3957214	4043082	4128146	4211994	4294107
Rio Grande do Sul	7768137	7879376	8007902	8136872	8265504	8393718	8520731	8645930	8768537
Mato Grosso do Sul	1368601	1401333	1440019	1478838	1517556	1556147	1594377	1632061	1668965
Mato Grosso do Sul	1136965	1207399	1291073	1375036	1458779	1542249	1624937	1706445	1786265
Goiás	3856828	3190172	3274754	3359628	3444280	3528656	3612242	3694635	3775322
Distrito Federal	1176084	1209734	1249677	1289758	1329734	1369580	1409053	1447962	1486065

1989	1990	1991	1992	1993	1994	1995	1996	1997	1998	1999
1014838	1069626	1132692	1150512	1241706	1291201	1339506	1225601	1251720	1272269	1292850
396335	406275	417718	429683	437499	446473	455253	482219	498787	512611	526448
1979597	2037078	2103243	2155090	2217585	2269569	2320229	2372927	2443776	2503454	2563197
192157	203977	217583	222939	241092	251792	262194	245394	252712	258874	265046
4665966	4798037	4950060	5051708	4717563	4825547	4930917	5488014	5627168	5744307	5861729
268432	278178	289397	305722	308792	317594	326186	377894	400250	419108	437972
886621	902074	919863	943205	970206	990753	1006991	1047059	1079126	1106142	1133185
4758722	4838464	4930253	4983237	5088909	5160987	5231256	5197493	5270433	5331511	5392688
2500745	2538583	2582137	2598639	2657442	2691614	2724982	2668382	2691217	2710164	2729367
6168566	6260651	6366647	6446971	6549838	6633257	6714296	6791080	6901814	6994628	7087614
2320531	2364712	2415567	2441504	2503421	2543505	2582305	2552123	2587684	2617762	2647689
3121959	3158757	3201114	3220056	3274408	3307528	3339959	3299228	3325290	3347113	3369056
6947006	7031080	7127855	7177026	7295110	7371163	7445215	7374243	7441760	7498540	7555392
2416472	2461858	2514100	2535713	2604393	2645421	2685400	2625264	2654979	2679966	2704954
1427265	1457302	1491876	1515856	1551598	1578785	1605253	1618388	1651423	1679086	1706825
11424650	11630752	11867991	11990115	12278010	12464546	12645885	12508000	12675542	12816674	12958000
15308819	15510734	15743152	15911713	16144803	16327334	16505371	16645734	16877709	17072771	17268278
2494583	2543877	2600618	2637248	2698670	2743241	2786656	2800275	2850653	2893030	2935504
12529211	12658679	12807706	12916221	13065268	13182302	13296442	13362400	13511248	13636604	13762121
30386139	30945297	31588925	32047865	32701245	33207158	33699405	34019366	34650594	35180936	35712140
8298219	8368181	8448713	8549328	8587850	8651101	8712805	8992261	9130460	9246886	9363597
4374098	4452150	4541994	4602398	4697194	4767955	4836588	4867657	4950589	5020437	5090495
8887976	9004520	9138670	9229120	9370552	9475775	9578691	9618765	9745978	9850773	9955239
1704916	1739995	1780373	1807102	1850145	1881840	1912841	1922994	1959634	1990545	2021508
1864023	1939896	2027231	2065042	2163293	2246789	2313648	2228678	2280526	2324170	2367958
3853923	3930620	4018903	4108982	4171509	4240736	4308541	4507903	4632463	4736749	4841133
1523184	1559403	1601094	1641125	1673152	1705889	1737813	1819398	1874390	1920714	1967112

2000	2001	2002	2003	2004	2005	2006	2007	2008	2009	2010
1379787	1407878	1431776	1455914	1479940	1534584	1562406	1590027	1493566	1503911	1562409
557526	574366	586945	600607	614205	669737	686650	703447	680073	691169	733559
2812557	2900218	2961804	3031079	3100136	3232319	3311046	3389081	3341096	3393357	3483985
324397	337253	346866	357296	367701	391318	403340	415281	412783	421497	450479
6192307	6341711	6453699	6574990	6695940	6970591	7110462	7249184	7321493	7431041	7581051
477032	498735	516514	534821	553100	594577	615724	636652	613164	626607	669526
1157098	1184855	1207008	1230188	1253256	1305708	1332443	1358889	1280509	1292063	1383445
5651475	5730432	5803283	5873646	5943807	6103338	6184543	6265102	6305539	6367111	6574789
2843278	2872983	2898191	2923695	2949133	3006886	3036271	3065488	3119697	3145164	3118360
7430661	7547684	7654540	7758437	7862067	8097290	8217140	8335849	8450527	8547750	8452381
2776782	2815203	2852800	2888087	2923287	3003040	3043740	3084106	3106430	3137646	3168027
3443825	3468534	3494965	3518607	3542167	3595849	3623198	3650180	3742606	3769954	3766528
7918344	8008255	8084722	8161828	8238849	8413601	8502602	8590868	8734194	8810318	8796448
2822621	2856563	2887526	2917678	2947717	3015901	3050649	3085053	3127557	3156101	3120494
1784475	1817318	1846042	1874597	1903065	1967818	2000768	2033430	1999374	2019755	2068017
13070250	13214146	13323150	13440544	13552649	13815260	13950125	14083771	14502575	14637500	14016906
17891494	18127024	18343518	18553335	18762405	19237434	19479262	19719285	19850072	20034068	19597330
3097232	3155048	3201712	3250205	3298541	3408360	3464280	3519712	3453648	3487094	3514952
14391282	14558561	14724479	14879144	15033317	15383422	15561720	15738536	15872362	16010386	15989929
37032403	37630105	38177734	38709339	39239362	40442820	41055761	41663568	41011635	41384089	41262199
9563458	9694769	9797965	9906812	10015425	10261840	10387408	10511933	10590169	10686228	10444526
5356360	5448702	5527718	5607160	5686503	5866590	5958295	6049251	6052587	6118727	6248436
10187798	10310021	10408428	10511009	10613256	10845002	10963216	11080317	10855214	10914042	10693929
2078001	2111030	2140620	2169704	2198640	2264489	2297994	2331243	2336058	2360550	2449024
2504353	2560537	2604723	2651313	2697717	2803272	2857024	2910255	2957732	3001725	3035122
5003228	5116395	5210366	5306424	5402335	5619919	5730762	5840650	5844996	5926308	6003788
2051146	2097450	2145838	2189792	2233614	2333109	2383784	2434033	2557158	2606884	2570160

2011	2012	2013	2014	2015	2016	2017	2018
1576455	1590011	1728214	1748531	1768204	1787279	1805788	1823741
746386	758786	776463	790101	803513	816687	829619	842290
3538387	3590985	3807921	3873743	3938336	4001667	4063614	4124033
460165	469524	488072	496936	505665	514229	522636	530879
7688593	7822205	7969654	8073924	8175113	8272724	8366628	8457229
684309	698602	734996	750912	766679	782295	797722	812961
1400892	1417694	1478164	1496880	1515126	1532902	1550194	1567016
6645761	6714314	6794301	6850884	6904241	6954036	7000229	7043339
3140328	3160748	3183404	3193956	3203262	3211411	3218485	3224536
8530155	8606005	8779338	8843553	8905225	8964432	9021232	9075744
3198657	3228198	3373959	3408510	3442175	3474998	3507003	3538218
3791315	3815171	3914421	3943885	3972202	3999415	4025558	4050662
8864906	8931028	9208550	9278152	9345603	9410772	9473707	9534634
3143384	3165472	3300935	3321305	3340502	3358527	3375382	3391142
2089819	2110867	2195662	2219574	2242937	2265779	2288116	2309961
14097534	14175341	15044137	15126371	15203934	15276566	15344447	15408073
19728701	19855332	20593356	20734097	20869101	20997560	21119536	21235870
3547055	3578067	3839366	3885049	3929911	3973697	4016356	4058079
16112678	16231365	16369179	16461173	16550024	16635996	16718956	16798421
41587182	41901219	43663669	44035304	44396484	44749699	45094866	45429330
10512349	10577755	10997465	11081692	11163018	11242720	11320892	11396262
6317054	6383286	6634254	6727148	6819190	6910553	7001161	7090682
10733030	10770603	11164043	11207274	11247972	11286500	11322895	11356804
2477542	2505088	2587269	2619657	2651235	2682386	2713147	2743142
3075936	3115336	3182113	3224357	3265486	3305531	3344544	3382487
6080716	6154996	6434048	6523222	6610681	6695855	6778772	6860047
2609998	2648532	2789761	2852372	2914830	2977216	3039444	3101220

Anexo II - Óbitos por Insuficiência Cardíaca, por Unidade da Federação - Brasil, 1980-2018.

Unidade da Federação	1980	1981	1982	1983	1984	1985	1986	1987	1988	1989
Rondônia	95	77	96	135	171	191	196	211	250	241
Acre	51	59	65	76	75	58	97	53	70	78
Amazonas	156	131	171	147	161	173	194	208	193	222
Roraima	16	10	37	15	11	14	12	19	17	15
Pará	570	572	566	543	643	655	657	575	701	633
Amapá	21	29	44	39	34	30	28	32	16	29
Tocantins										120
Maranhão	257	244	274	357	388	438	349	363	412	371
Piauí	188	207	209	251	344	331	370	299	365	322
Ceará	472	503	597	577	690	1040	721	545	638	711
Rio Grande do Norte	219	261	223	252	300	245	256	261	279	224
Paraíba	505	592	687	643	699	655	652	486	465	470
Pernambuco	1146	1071	1236	1028	1043	996	1138	1074	1211	1177
Alagoas	396	443	450	417	343	271	304	281	347	395
Sergipe	195	218	186	199	196	220	245	236	305	197
Bahia	1472	1553	1651	1591	1682	1700	1790	1641	1896	1921
Minas Gerais	4852	5074	4820	4823	5192	5235	5062	4621	5123	5122
Espírito Santo	528	541	564	513	529	634	515	507	603	585
Rio de Janeiro	3678	4402	4145	4639	4614	4909	4742	4946	5325	5087
São Paulo	9305	8916	8713	8666	8712	8644	8380	8265	9129	8664
Paraná	2326	2666	2897	2725	2575	2125	2052	1876	2156	1751
Santa Catarina	1207	1126	1148	1165	1267	1043	964	945	1055	953
Rio Grande do Sul	3136	2758	2555	3021	3138	2774	2903	2802	3618	3558
Mato Grosso do Sul	261	299	251	365	375	367	427	352	392	393
Mato Grosso do Sul	132	165	162	204	251	250	259	278	318	245
Goiás	725	824	759	761	903	853	871	842	1018	837
Distrito Federal	128	135	164	199	263	280	247	208	228	216

1990	1991	1992	1993	1994	1995	1996	1997	1998	1999	2000
243	248	217	276	157	197	196	148	172	201	182
61	91	55	74	86	96	83	73	68	62	55
176	139	136	174	214	236	145	158	158	180	171
21	16	24	7	12	10	19	11	9	8	15
736	561	723	770	713	634	547	546	597	616	555
40	28	33	26	30	32	29	47	35	27	40
73	96	129	130	135	153	151	160	170	168	163
398	363	425	399	412	443	309	407	439	462	532
362	283	380	411	353	313	257	311	328	322	390
823	852	803	847	906	826	853	832	942	881	896
245	271	299	344	409	333	282	249	298	320	258
463	527	431	510	524	544	461	517	474	480	511
1158	1165	1258	1374	1501	1450	1333	1289	1276	1162	1181
310	435	402	407	351	398	370	452	502	410	391
262	166	258	275	185	111	166	159	156	211	200
1837	1906	1995	2122	2144	2194	1917	1782	1910	1891	1920
4993	4908	4749	5107	5032	4549	4489	4168	4292	3989	3846
576	596	555	696	586	579	557	436	535	474	506
4819	4489	4223	4886	4534	4087	3944	3495	3286	2972	2617
8527	7636	7895	8360	8325	7917	8393	7913	7676	7079	6415
1977	1846	1877	2109	2190	2244	2401	2420	2467	2228	2288
989	820	963	964	865	911	948	920	951	902	936
3125	2949	3134	3184	2914	3621	3029	2540	2791	2470	2264
399	312	331	373	493	420	485	474	478	480	370
241	308	319	390	357	352	291	379	376	351	359
781	1004	1154	1161	1339	1082	990	1065	1044	939	927
215	217	188	175	145	163	231	286	199	131	164

2001	2002	2003	2004	2005	2006	2007	2008	2009	2010	2011
221	208	172	163	155	183	175	155	197	210	198
70	68	68	57	97	80	63	60	72	68	76
185	159	171	178	185	159	177	196	183	176	174
9	13	22	25	23	17	33	31	24	45	40
600	517	571	520	582	580	653	656	596	618	733
39	43	34	34	22	23	35	31	29	33	39
208	181	208	184	206	204	205	163	169	177	188
522	621	555	542	665	733	730	740	713	664	750
498	510	539	522	514	563	573	567	466	436	418
893	909	921	865	797	1073	890	953	933	823	895
256	336	269	338	337	374	406	393	414	403	482
524	537	595	675	667	709	745	721	777	772	753
1120	1117	1170	1170	1106	1170	1170	1040	1011	953	934
489	437	534	574	513	481	445	493	450	444	488
210	197	184	246	219	169	217	205	202	194	165
1864	1994	1935	1961	1722	2039	2070	1924	1859	1761	1946
3588	3488	3644	3794	3421	3674	3601	3375	3405	3645	3683
519	455	429	407	392	478	451	431	377	444	240
2671	2780	2540	2592	2348	2557	2495	2490	2392	2452	2438
5917	5557	5680	5762	6068	5881	6013	5982	6193	6267	6321
2110	2172	2101	2089	1954	1986	2001	1962	1853	1867	1933
921	846	870	947	879	846	838	883	855	1023	1046
2162	2190	2030	1987	1918	2022	2155	2108	2186	2127	2110
338	330	351	319	287	293	287	287	367	297	140
404	354	328	326	363	395	367	390	373	367	328
926	1014	968	929	882	974	944	967	909	947	983
181	214	236	335	236	260	239	335	294	291	286

2012	2013	2014	2015	2016	2017	2018
173	174	158	141	193	171	199
53	68	65	93	82	77	75
175	215	232	227	255	235	238
51	37	41	32	31	23	32
680	608	663	714	760	663	719
39	26	42	44	40	47	45
156	182	166	133	143	108	110
718	740	732	674	762	797	766
482	477	461	465	350	411	428
899	930	1166	1268	1182	1170	1094
409	408	377	349	465	340	382
719	768	725	683	721	656	698
909	973	867	893	975	840	816
427	401	411	403	434	381	370
164	173	186	186	161	153	158
1858	1856	1894	1876	1902	1858	1913
3567	3431	3443	3431	3606	3618	3550
165	145	144	174	199	189	194
2396	2647	2481	2593	2904	2964	2818
6175	6340	6034	6501	6878	6752	6399
1937	1959	1982	2059	2121	1874	1518
940	1059	932	991	1131	916	967
1820	1897	1802	1779	1897	1793	1623
101	151	163	182	213	140	127
310	279	287	304	326	288	317
1013	1036	1018	960	883	872	809
331	289	284	259	146	113	101

Anexo III - Taxa de mortalidade por Insuficiência Cardíaca, por 100.000 habitantes, por Unidade da Federação - Brasil 1980-2018.

Unidade da Federação	1980	1981	1982	1983	1984	1985	1986	1987	1988
Rondônia	19,37	14,24	15,97	20,40	23,68	24,41	23,27	23,42	26,08
Acre	16,95	19,01	20,23	22,87	21,85	16,38	26,57	14,11	18,13
Amazonas	10,92	8,84	11,06	9,14	9,63	9,97	10,79	11,18	10,05
Roraima	20,24	11,13	35,96	12,93	8,53	9,86	7,75	11,34	9,44
Pará	16,77	16,24	15,43	14,23	16,24	15,95	15,46	13,09	15,47
Amapá	11,99	15,75	22,58	18,97	15,71	13,21	11,78	12,89	6,19
Tocantins									
Maranhão	6,44	6,00	6,59	8,41	8,95	9,91	7,74	7,90	8,81
Piauí	8,80	9,52	9,44	11,12	14,97	14,14	15,54	12,34	14,83
Ceará	8,93	9,36	10,91	10,35	12,15	18,00	12,27	9,12	10,50
Rio Grande do Norte	11,55	13,47	11,22	12,38	14,39	11,49	11,74	11,71	12,26
Paraíba	18,25	21,12	24,16	22,29	23,89	22,08	21,69	15,96	15,08
Pernambuco	18,67	17,22	19,58	16,05	16,05	15,11	17,03	15,86	17,65
Alagoas	20,00	21,89	21,70	19,63	15,78	12,19	13,37	12,10	14,64
Sergipe	17,12	18,68	15,49	16,13	15,47	16,92	18,38	17,29	21,84
Bahia	15,59	16,11	16,73	15,76	16,29	16,11	16,61	14,92	16,91
Minas Gerais	36,29	37,42	34,97	34,43	36,49	36,22	34,50	31,04	33,92
Espírito Santo	26,12	26,16	26,57	23,57	23,71	27,74	22,02	21,19	24,67
Rio de Janeiro	32,62	38,58	35,88	39,67	38,98	40,98	39,12	40,34	42,96
São Paulo	37,18	34,90	33,30	32,36	31,80	30,86	29,28	28,28	30,62
Paraná	30,51	34,66	37,28	34,73	32,49	26,56	25,40	23,01	26,21
Santa Catarina	33,30	30,44	30,33	30,10	32,02	25,80	23,35	22,44	24,57
Rio Grande do Sul	40,37	35,00	31,91	37,13	37,97	33,05	34,07	32,41	41,26
Mato Grosso do Sul	19,07	21,34	17,43	24,68	24,71	23,58	26,78	21,57	23,49
Mato Grosso do Sul	11,61	13,67	12,55	14,84	17,21	16,21	15,94	16,29	17,80
Goiás	18,80	25,83	23,18	22,65	26,22	24,17	24,11	22,79	26,96
Distrito Federal	10,88	11,16	13,12	15,43	19,78	20,44	17,53	14,37	15,34

1989	1990	1991	1992	1993	1994	1995	1996	1997	1998	1999
23,75	22,72	21,89	18,86	22,23	12,16	14,71	15,99	11,82	13,52	15,55
19,68	15,01	21,79	12,80	16,91	19,26	21,09	17,21	14,64	13,27	11,78
11,21	8,64	6,61	6,31	7,85	9,43	10,17	6,11	6,47	6,31	7,02
7,81	10,30	7,35	10,77	2,90	4,77	3,81	7,74	4,35	3,48	3,02
13,57	15,34	11,33	14,31	16,32	14,78	12,86	9,97	9,70	10,39	10,51
10,80	14,38	9,68	10,79	8,42	9,45	9,81	7,67	11,74	8,35	6,16
13,53	8,09	10,44	13,68	13,40	13,63	15,19	14,42	14,83	15,37	14,83
7,80	8,23	7,36	8,53	7,84	7,98	8,47	5,95	7,72	8,23	8,57
12,88	14,26	10,96	14,62	15,47	13,11	11,49	9,63	11,56	12,10	11,80
11,53	13,15	13,38	12,46	12,93	13,66	12,30	12,56	12,05	13,47	12,43
9,65	10,36	11,22	12,25	13,74	16,08	12,90	11,05	9,62	11,38	12,09
15,05	14,66	16,46	13,38	15,58	15,84	16,29	13,97	15,55	14,16	14,25
16,94	16,47	16,34	17,53	18,83	20,36	19,48	18,08	17,32	17,02	15,38
16,35	12,59	17,30	15,85	15,63	13,27	14,82	14,09	17,02	18,73	15,16
13,80	17,98	11,13	17,02	17,72	11,72	6,91	10,26	9,63	9,29	12,36
16,81	15,79	16,06	16,64	17,28	17,20	17,35	15,33	14,06	14,90	14,59
33,46	32,19	31,18	29,85	31,63	30,82	27,56	26,97	24,70	25,14	23,10
23,45	22,64	22,92	21,04	25,79	21,36	20,78	19,89	15,29	18,49	16,15
40,60	38,07	35,05	32,70	37,40	34,39	30,74	29,52	25,87	24,10	21,60
28,51	27,56	24,17	24,64	25,56	25,07	23,49	24,67	22,84	21,82	19,82
21,10	23,63	21,85	21,95	24,56	25,31	25,76	26,70	26,50	26,68	23,79
21,79	22,21	18,05	20,92	20,52	18,14	18,84	19,48	18,58	18,94	17,72
40,03	34,70	32,27	33,96	33,98	30,75	37,80	31,49	26,06	28,33	24,81
23,05	22,93	17,52	18,32	20,16	26,20	21,96	25,22	24,19	24,01	23,74
13,14	12,42	15,19	15,45	18,03	15,89	15,21	13,06	16,62	16,18	14,82
21,72	19,87	24,98	28,08	27,83	31,57	25,11	21,96	22,99	22,04	19,40
14,18	13,79	13,55	11,46	10,46	8,50	9,38	12,70	15,26	10,36	6,66

2000	2001	2002	2003	2004	2005	2006	2007	2008	2009	2010
13,19	15,70	14,53	11,81	11,01	10,10	11,71	11,01	10,38	13,10	13,44
9,87	12,19	11,59	11,32	9,28	14,48	11,65	8,96	8,82	10,42	9,27
6,08	6,38	5,37	5,64	5,74	5,72	4,80	5,22	5,87	5,39	5,05
4,62	2,67	3,75	6,16	6,80	5,88	4,21	7,95	7,51	5,69	9,99
8,96	9,46	8,01	8,68	7,77	8,35	8,16	9,01	8,96	8,02	8,15
8,39	7,82	8,33	6,36	6,15	3,70	3,74	5,50	5,06	4,63	4,93
14,09	17,55	15,00	16,91	14,68	15,78	15,31	15,09	12,73	13,08	12,79
9,41	9,11	10,70	9,45	9,12	10,90	11,85	11,65	11,74	11,20	10,10
13,72	17,33	17,60	18,44	17,70	17,09	18,54	18,69	18,17	14,82	13,98
12,06	11,83	11,88	11,87	11,00	9,84	13,06	10,68	11,28	10,92	9,74
9,29	9,09	11,78	9,31	11,56	11,22	12,29	13,16	12,65	13,19	12,72
14,84	15,11	15,36	16,91	19,06	18,55	19,57	20,41	19,26	20,61	20,50
14,91	13,99	13,82	14,34	14,20	13,15	13,76	13,62	11,91	11,48	10,83
13,85	17,12	15,13	18,30	19,47	17,01	15,77	14,42	15,76	14,26	14,23
11,21	11,56	10,67	9,82	12,93	11,13	8,45	10,67	10,25	10,00	9,38
14,69	14,11	14,97	14,40	14,47	12,46	14,62	14,70	13,27	12,70	12,56
21,50	19,79	19,01	19,64	20,22	17,78	18,86	18,26	17,00	17,00	18,60
16,34	16,45	14,21	13,20	12,34	11,50	13,80	12,81	12,48	10,81	12,63
18,18	18,35	18,88	17,07	17,24	15,26	16,43	15,85	15,69	14,94	15,33
17,32	15,72	14,56	14,67	14,68	15,00	14,32	14,43	14,59	14,96	15,19
23,92	21,76	22,17	21,21	20,86	19,04	19,12	19,04	18,53	17,34	17,88
17,47	16,90	15,30	15,52	16,65	14,98	14,20	13,85	14,59	13,97	16,37
22,22	20,97	21,04	19,31	18,72	17,69	18,44	19,45	19,42	20,03	19,89
17,81	16,01	15,42	16,18	14,51	12,67	12,75	12,31	12,29	15,55	12,13
14,34	15,78	13,59	12,37	12,08	12,95	13,83	12,61	13,19	12,43	12,09
18,53	18,10	19,46	18,24	17,20	15,69	17,00	16,16	16,54	15,34	15,77
8,00	8,63	9,97	10,78	15,00	10,12	10,91	9,82	13,10	11,28	11,32

2011	2012	2013	2014	2015	2016	2017	2018
12,56	10,88	10,07	9,04	7,97	10,80	9,47	10,91
10,18	6,98	8,76	8,23	11,57	10,04	9,28	8,90
4,92	4,87	5,65	5,99	5,76	6,37	5,78	5,77
8,69	10,86	7,58	8,25	6,33	6,03	4,40	6,03
9,53	8,69	7,63	8,21	8,73	9,19	7,92	8,50
5,70	5,58	3,54	5,59	5,74	5,11	5,89	5,54
13,42	11,00	12,31	11,09	8,78	9,33	6,97	7,02
11,29	10,69	10,89	10,68	9,76	10,96	11,39	10,88
13,31	15,25	14,98	14,43	14,52	10,90	12,77	13,27
10,49	10,45	10,59	13,18	14,24	13,19	12,97	12,05
15,07	12,67	12,09	11,06	10,14	13,38	9,69	10,80
19,86	18,85	19,62	18,38	17,19	18,03	16,30	17,23
10,54	10,18	10,57	9,34	9,56	10,36	8,87	8,56
15,52	13,49	12,15	12,37	12,06	12,92	11,29	10,91
7,90	7,77	7,88	8,38	8,29	7,11	6,69	6,84
13,80	13,11	12,34	12,52	12,34	12,45	12,11	12,42
18,67	17,96	16,66	16,61	16,44	17,17	17,13	16,72
6,77	4,61	3,78	3,71	4,43	5,01	4,71	4,78
15,13	14,76	16,17	15,07	15,67	17,46	17,73	16,78
15,20	14,74	14,52	13,70	14,64	15,37	14,97	14,09
18,39	18,31	17,81	17,89	18,44	18,87	16,55	13,32
16,56	14,73	15,96	13,85	14,53	16,37	13,08	13,64
19,66	16,90	16,99	16,08	15,82	16,81	15,84	14,29
5,65	4,03	5,84	6,22	6,86	7,94	5,16	4,63
10,66	9,95	8,77	8,90	9,31	9,86	8,61	9,37
16,17	16,46	16,10	15,61	14,52	13,19	12,86	11,79
10,96	12,50	10,36	9,96	8,89	4,90	3,72	3,26

Anexo IV - Mortalidade proporcional por Insuficiência Cardíaca, por Unidade da Federação - Brasil, 1980-2018.

Unidade da Federação	1980	1981	1982	1983	1984	1985	1986	1987	1988
Rondônia	2,94	2,57	3,04	3,46	3,79	4,05	3,57	3,81	4,59
Acre	3,58	3,95	4,38	4,80	4,32	3,50	4,70	2,55	3,26
Amazonas	2,45	2,11	2,58	2,13	2,14	2,35	2,55	2,66	2,48
Roraima	4,06	2,08	6,27	2,70	1,80	2,14	1,74	2,62	2,21
Pará	3,45	3,50	3,54	3,16	3,50	3,66	3,51	3,17	3,84
Amapá	2,68	3,05	5,35	4,49	3,72	3,08	2,47	3,27	1,51
Tocantins									
Maranhão	3,84	3,53	2,52	2,44	2,38	3,04	2,36	2,80	2,84
Piauí	3,53	3,56	3,00	3,11	4,26	3,57	4,18	3,57	4,36
Ceará	2,63	2,57	3,16	2,26	2,55	4,45	3,22	2,56	2,78
Rio Grande do Norte	2,07	2,59	2,53	2,77	3,06	2,54	2,81	2,53	2,66
Paraíba	2,04	2,44	3,09	2,85	2,80	3,18	2,93	2,31	2,30
Pernambuco	1,98	1,93	2,33	1,89	1,73	1,82	1,97	2,00	2,12
Alagoas	2,27	2,52	2,57	2,41	1,77	1,64	1,72	1,70	2,04
Sergipe	2,62	2,80	2,48	2,80	2,50	2,94	3,22	3,36	4,29
Bahia	3,24	3,22	3,38	3,19	3,15	3,39	3,44	3,19	3,73
Minas Gerais	5,14	5,47	5,33	5,37	5,64	5,59	5,42	5,14	5,49
Espírito Santo	4,10	4,21	4,29	3,77	3,94	4,53	3,66	3,71	4,25
Rio de Janeiro	3,97	4,77	4,63	4,96	4,79	5,03	4,71	4,88	4,95
São Paulo	5,30	5,07	4,98	4,87	4,69	4,74	4,43	4,35	4,53
Paraná	4,88	5,78	6,50	5,90	5,54	4,61	4,39	4,11	4,54
Santa Catarina	6,11	5,85	6,13	5,78	6,13	5,14	4,49	4,31	4,50
Rio Grande do Sul	6,00	5,47	5,08	5,75	5,72	5,19	5,33	5,01	6,16
Mato Grosso do Sul	3,65	3,93	3,67	4,75	4,79	4,59	5,23	4,29	4,64
Mato Grosso do Sul	4,05	4,48	4,22	4,23	4,49	4,11	3,99	4,09	4,44
Goiás	4,44	4,39	3,78	3,83	4,49	4,01	4,03	3,90	4,61
Distrito Federal	2,32	2,46	3,12	3,77	4,92	5,09	4,40	3,69	3,92

1989	1990	1991	1992	1993	1994	1995	1996	1997	1998	1999
4,40	4,38	5,12	5,03	5,78	3,30	3,74	4,28	3,06	3,13	3,73
4,39	3,99	4,72	2,67	3,48	3,76	4,42	3,67	3,21	2,85	2,93
2,84	2,20	2,04	1,91	2,26	2,56	2,79	1,82	1,92	1,82	1,89
1,96	2,46	1,96	3,13	0,90	1,29	1,03	1,85	1,19	0,82	0,63
3,91	3,99	3,29	4,27	4,29	3,92	3,62	3,19	2,96	2,96	2,91
2,88	4,17	2,51	3,05	2,11	2,04	2,12	1,82	2,84	2,28	1,59
4,95	5,16	3,89	4,88	4,33	4,85	4,70	4,07	4,31	4,15	3,96
2,83	2,93	2,72	3,13	2,88	3,34	3,61	2,60	3,05	2,90	3,22
4,15	4,79	4,60	4,84	4,87	4,35	4,11	3,82	4,04	3,95	3,74
3,07	3,73	3,72	3,52	3,08	3,15	2,94	2,93	2,69	2,98	2,54
2,30	2,90	2,66	2,88	2,89	3,57	2,65	2,31	2,08	2,39	2,54
2,49	2,50	2,94	2,53	2,66	2,88	2,93	2,61	2,98	2,68	2,80
2,24	2,25	2,35	2,58	2,68	3,03	3,07	2,76	2,61	2,46	2,25
2,61	2,06	3,07	2,80	2,79	2,51	2,74	2,68	3,17	3,04	2,87
2,66	3,62	2,07	2,78	3,23	2,20	1,14	1,88	1,90	1,69	2,25
3,80	3,83	3,83	3,84	4,08	3,97	4,15	3,51	3,40	3,42	3,24
5,61	5,53	5,49	5,03	5,21	5,20	4,81	4,64	4,26	4,37	4,02
4,40	3,99	4,06	3,77	4,28	3,67	3,68	3,33	2,75	3,05	2,74
4,75	4,47	4,26	3,97	4,29	3,89	3,51	3,35	3,09	2,85	2,62
4,31	4,13	3,85	3,88	3,86	3,74	3,47	3,57	3,40	3,31	2,99
3,71	4,02	3,97	3,86	4,10	4,19	4,37	4,45	4,53	4,42	4,07
4,16	4,21	3,68	4,10	3,83	3,43	3,44	3,42	3,41	3,44	3,24
6,18	5,31	5,08	5,20	4,94	4,58	5,57	4,48	3,95	4,02	3,67
4,50	4,55	3,64	3,60	3,90	4,73	3,93	4,36	4,30	4,34	4,18
3,57	3,72	4,32	4,34	5,08	4,49	3,94	3,48	4,15	3,69	3,26
4,48	5,10	5,61	5,81	5,66	6,11	4,78	4,61	4,62	4,52	3,98
3,45	3,21	3,17	2,71	2,29	1,80	2,01	2,81	3,40	2,37	1,53

2000	2001	2002	2003	2004	2005	2006	2007	2008	2009	2010
3,25	3,68	3,46	2,73	2,58	2,49	3,02	3,16	2,47	2,88	2,91
2,15	2,81	2,74	2,68	2,13	3,45	2,90	2,25	2,10	2,49	2,26
1,64	1,76	1,50	1,62	1,63	1,66	1,37	1,51	1,54	1,41	1,32
1,13	0,75	1,04	1,63	1,78	1,60	1,13	2,21	2,10	1,56	2,75
2,58	2,53	2,24	2,27	2,01	2,21	2,15	2,37	2,22	1,95	1,96
2,38	1,99	2,17	1,79	1,75	1,25	1,28	1,86	1,47	1,36	1,52
3,73	4,34	3,76	4,19	3,58	4,08	3,99	3,66	2,78	2,78	2,73
3,32	2,64	2,89	2,47	2,38	2,78	3,24	3,07	2,91	2,77	2,55
3,37	4,00	3,91	3,89	3,84	3,61	3,89	3,85	3,66	2,99	2,79
2,68	2,50	2,33	2,27	2,11	2,00	2,63	2,20	2,21	2,11	1,88
2,01	1,93	2,37	1,96	2,31	2,38	2,74	2,79	2,52	2,55	2,51
2,80	2,78	2,73	2,98	3,27	3,29	3,52	3,50	3,20	3,41	3,30
2,25	2,16	2,14	2,21	2,19	2,12	2,26	2,20	1,92	1,85	1,75
2,73	3,17	2,76	3,50	3,65	3,35	3,12	2,68	3,01	2,64	2,50
2,11	2,16	2,00	1,91	2,57	2,24	1,76	2,23	2,00	1,88	1,77
3,23	3,06	3,15	3,02	3,03	2,66	3,02	3,07	2,71	2,53	2,31
4,00	3,66	3,57	3,48	3,54	3,22	3,29	3,23	2,99	2,98	3,02
2,87	2,94	2,58	2,33	2,19	2,10	2,43	2,30	2,11	1,85	2,09
2,36	2,35	2,38	2,19	2,21	2,05	2,16	2,08	2,03	1,93	1,92
2,68	2,51	2,34	2,36	2,36	2,57	2,41	2,46	2,40	2,41	2,37
4,08	3,83	3,82	3,65	3,47	3,32	3,28	3,25	3,08	2,85	2,79
3,29	3,26	2,94	2,95	3,10	2,94	2,78	2,61	2,77	2,53	2,97
3,33	3,17	3,16	2,88	2,75	2,69	2,79	2,86	2,85	2,85	2,73
3,34	3,02	2,89	2,89	2,54	2,29	2,30	2,17	2,17	2,70	2,05
3,13	3,49	2,91	2,69	2,53	2,81	3,05	2,87	2,81	2,62	2,45
4,00	3,87	3,96	3,77	3,37	3,29	3,58	3,35	3,21	2,98	2,90
1,92	2,07	2,36	2,60	3,49	2,51	2,73	2,42	3,26	2,81	2,68

2011	2012	2013	2014	2015	2016	2017	2018
2,83	2,28	2,32	2,07	1,77	2,31	2,08	2,44
2,41	1,61	2,05	1,87	2,65	2,18	2,01	1,84
1,22	1,18	1,42	1,46	1,36	1,52	1,36	1,34
2,50	2,87	1,90	2,10	1,53	1,44	0,94	1,15
2,25	2,01	1,78	1,87	1,91	1,97	1,66	1,78
1,56	1,50	0,97	1,47	1,49	1,34	1,49	1,35
2,83	2,35	2,65	2,33	1,80	1,91	1,34	1,41
2,59	2,49	2,39	2,28	2,00	2,22	2,26	2,22
2,45	2,76	2,65	2,48	2,40	1,83	2,07	2,14
1,87	1,85	1,80	2,25	2,30	2,18	1,97	1,92
2,71	2,28	2,16	1,97	1,73	2,12	1,59	1,80
3,06	2,86	2,96	2,87	2,59	2,57	2,43	2,62
1,63	1,59	1,67	1,50	1,43	1,46	1,31	1,32
2,62	2,33	2,09	2,16	2,04	2,09	1,84	1,91
1,45	1,39	1,42	1,52	1,38	1,19	1,15	1,21
2,50	2,32	2,28	2,27	2,16	2,16	2,05	2,12
3,00	2,85	2,73	2,70	2,62	2,67	2,62	2,62
1,12	0,76	0,67	0,65	0,78	0,87	0,78	0,83
1,92	1,90	2,04	1,90	1,96	2,06	2,17	2,00
2,34	2,28	2,29	2,14	2,26	2,32	2,29	2,15
2,82	2,82	2,80	2,86	2,91	2,84	2,62	2,06
2,91	2,65	2,92	2,52	2,61	2,81	2,29	2,34
2,63	2,29	2,29	2,19	2,16	2,17	2,08	1,83
0,98	0,69	1,01	1,08	1,18	1,27	0,88	0,77
2,15	1,95	1,73	1,69	1,78	1,86	1,63	1,74
2,88	2,82	2,86	2,70	2,47	2,32	2,18	2,05
2,54	2,93	2,53	2,36	2,16	1,21	0,90	0,83
