# Supplementary material for: Mortalidade por Insuficiência Cardíaca e Desenvolvimento Socioeconômico no Brasil, 1980 a 2018
Source: Arq Bras Cardiol. 2021 Sep 16;117(5):944–51. [Article in Portuguese] doi: 10.36660/abc.20200902 (PMC8682101; doi:10.36660/abc.20200902)
Supplement: Translation

# Translation: Mortality Due to Heart Failure and Socioeconomic Development in Brazil between 1980 and 2018

**Authors:** Sonia Carvalho Santos, Paolo Blanco Villela, Gláucia Maria Moraes de Oliveira

**Affiliations:** 1 Brazil Universidade Federal do Rio de Janeiro – Cardiologia, Rio de Janeiro, RJ – Brazil

**Keywords:** Heart Failure, Development Indicators, Mortality Registries

## Abstract

**Background:**

Studies on mortality from heart failure (HF) in Brazil and in the country’s Geographic Regions (GRs) are scarce.

**Objective:**

To analyze the temporal progression of HF mortality rates by sex and age group in Brazil and its GRs and Federative Units (FUs) from 1980 to 2018, and the associations between mortality rates at each FU and the Municipal Human Development Index (MHDI).

**Methods:**

Time series analysis of deaths due to HF categorized by sex and age groups in Brazil and Brazilian GRs and FUs from 1980 to 2018. Death and population data were obtained from the DATASUS for estimation of crude and standardized mortality rates per 100,000 inhabitants (direct method, Brazilian population in the year 2000). We calculated the 3-year moving averages of the standardized rates. The MHDIs of the FUs in 1991 and 2010 were obtained from Atlas Brasil and were correlated with mortality rates using Pearson’s correlation at a 5% significance level.

**Results:**

Mortality due to HF decreased in Brazil after 2008, reaching a similar level at the end of 2018 in the GRs and FUs, and was higher in men during almost all periods and age groups, except for those over the age of 60 years after 1995 in the South region. There was an inverse relationship between MHDI and reduction in mortality rates (0.73).

**Conclusion:**

There was a progressive reduction in mortality rates due to HF in Brazil from 2008 to 2018, with similar levels in 2018 in the GRs and FUs and higher rates in men. These reductions appear to be related more to the 2010 MHDI than the percentage increase over time.

## Introduction

Annual mortality rates are higher for cardiovascular diseases (CVDs) than any other cause, making these the first causes of death worldwide. About 17.9 million people are estimated to have died from CVDs in 2016, representing 31% of all global deaths and more than three-quarters of CVD deaths in low- and middle-income countries.^1^ Among all CVDs, heart failure (HF) stands out for its high and increasing morbidity and mortality rates.^2^

Mortality rates due to HF are not estimated in the Global Burden of Disease (GBD) data since this condition is considered to be the common end to several diseases (also known as garbage code, *i.e.* , a nonspecific, incomplete code that does not identify clearly the underlying cause of death)^3^ and redistributes the deaths by the conditions that were responsible for their occurrence. According to the GBD, the prevalence and standardized rates of HF per 100,000 inhabitants in Brazil were 670,194.8 (95% uncertainty interval [UI] = 589,952.6; 753,672.6) and 818.1 (95% UI = 718.1; 922.8) in 1990 and 1,686,320.1 (95% UI = 1,478,563.8; 1,890,537.3) and 777.2 (95% UI = 680.0; 874.80) in 2017, with a percentage reduction of -5% (95% UI = -7.1; -3) in the standardized prevalence rate over 27 years.^3^

Studies on HF mortality in Brazil based on data from the Mortality Information System in Geographic Regions (GRs) and Federative Units (FUs) with different levels of socioeconomic development are scarce. A study on underlying causes of death carried out between 2004 and 2011 observed that the proportional mortality due to HF increased with age, and that the highest percentages in Brazil and its GRs were observed among elderly women.^4^ The authors found ischemic heart disease to be the most frequent cause for the occurrence and development of HF, and suggested that regional differences may be a consequence of socioeconomic conditions and health care structures, among other factors.^5^ A recent study conducted between 2008 and 2015 in Paraíba, the state with the lowest socioeconomic development, has reported that the HF mortality in absolute numbers had a nonsignificant decline between 2008 and 2015 (R = -0.513), and that the same happened in Brazil (R = -0.412), with no observed statistically significant differences regarding sex and age groups.^6^ However, the relationships of HF and social and economic indicators in Brazil have not been much explored in the literature.

The Human Development Index (HDI) is a summary measure of the population's health, education, and income that evaluates long and healthy life, access to knowledge, and standard of living. This index appears to be a good socioeconomic indicator to assess the complex relationships between social determinants and CVD. Since 2009, the HDI has been composed of the life expectancy at birth, average years of schooling for the adult population and expected years of schooling for children (school enrollment rate), and the gross national income per capita.^7^

The present study aimed to analyze the temporal evolution of HF mortality rates according to sex and age group in Brazil and in the country's GRs and FUs over the last 39 years, and the associations of the mortality rates with the MHDI, an index chosen to compare the socioeconomic development among the FUs.

## Materials and Methods

This was an ecological and descriptive study of historical series of death certificates related to deaths due to HF that occurred in Brazil between 1980 and 2018, across all age groups and in both sexes.

Information on the underlying cause of death was retrieved from the website of the Brazilian Mortality Information System ( *Sistema de Informação de Mortalidade* , SIM) of the Department of Informatics of the Unified Health System (DATASUS) of the Ministry of Health.^8^ After downloading the database, the original files in a .CSV format were converted into a .XLS format using the program Microsoft Excel,^9^ which was also used for the data analysis and construction of graphs and tables. For identification of deaths with HF as the underlying cause, we used the categories 428 of the ICD-9^10^ for deaths that occurred between 1980 and 1995, and I50 of the ICD-10^11^ for deaths that occurred after 1996.

Information on the resident population was also retrieved from the DATASUS website,^8^ which in turn considered the census data from the Brazilian Institute of Geography and Statistics ( *Instituto Brasileiro de Geografia e Estatística* , IBGE) from 1980, 1991, 2000, and 2010, intercensal projections until 2012, and population projections from 2013 onwards.

The annual crude and standardized mortality rates in the FUs per 100,000 inhabitants were estimated by the direct method,^12^ using as standard the age structure of the Brazilian population in 2000. For each FU, we calculated the moving averages of the standardized rates every 3 years, disregarding the initial 2 years of the series (1980 and 1981 for all FUs; 1989 and 1990 for Tocantins) until 2018. The FUs were grouped into their GRs (North, Northeast, Southeast, South, and Midwest). Of note, after 1989, the North region started incorporating data from Tocantins, a FU created in 1988.

We estimated the crude mortality rates by GR in three age groups (up to 29 years, 30–59 years, and 60 years or more) over seven periods of 5 years and in a period of 4 years (2015 to 2018), with subsequent calculation of the ratio rates of men/women.

The MHDI of each FU corresponding to the years 1991 and 2010 were obtained from the website Atlas Brasil.^13^ The data result from adapting the calculation of the country's global HDI to municipal and state levels, carried out by the United Nations Development Programme (UNDP - Brazil), by the Institute for Applied Economic Research (IPEA), and by the João Pinheiro Foundation, thus creating the Municipal HDI (MHDI), which can be interpreted the same way as the global HDI but at a municipal and state level. Next, we calculated the percentage variation of the MHDI for each FU between 1991 and 2010, and its correlation with the percentage variation of the standardized mortality rates in the respective FUs between 1990 and 2018 using Pearson's correlation coefficient, adopting as significant a value below 0.05. Of note, we chose the year 1990 in this case for the beginning of the temporal series so that all FUs could be evaluated with the same time interval, considering the incorporation of Tocantins in 1988. We correlated the 2010 MHDI with the percentage variation of the standardized mortality rates in the respective FUs between 1990 and 2018.

## Results

We found 1,185,120 deaths between 1980 and 2018, of which 49.3% (584,155) occurred in men. Regarding the distribution of the deaths by GR, 48,533 occurred in the North, 245,898 in the Northeast, 602,105 in the Southeast, 218,496 in the South, and 70,088 in the Midwest. The complete data used for the study are available in Supplemental Tables 1 , 2 , 3 , and 4 .

Figure 1 shows the 3-year moving averages of the mortality rates standardized by age per 100,000 inhabitants at each FU grouped into the five GRs ( Figures 1A to 1E ) and the overall national rates ( Figure 1F ) between 1982 and 2018. In the North region, with the exception of Rondônia and Acre, in which the averages increased in the first and second decades of observation, respectively, all other FUs showed a progressive decline and, after 2008, the averages were similar in all FUs, with small oscillations until 2018 ( Figure 1A ). Since Tocantins was incorporated in 1988, this state's data are presented from 1989 onwards, and in this case, the calculation of the moving averages began after 1991 ( Figure 1A ). In the Northeast region ( Figure 1B ), Alagoas had the highest averages at the beginning of the period and, despite the downward trend, showed increases between 1998 and 2008, a similar trend to that observed in Piauí. Following the same trend of the North region, the averages of all FUs in the Northeast region after 2008 were similar to each other, showing the same evolutionary trend in the last 10 years of observation.

**Figure 1:** – *Shows the 3-year moving averages of the mortality rates standardized by age per 100,000 inhabitants at each FU grouped into the five GRs, (A- North, B-Northeast, C- Southeast, D- South, E-Midwest and the national total (F).*

The FUs of the Southeast region ( Figure 1C ) showed increased averages at the beginning of the period followed by a progressive decline over the years, especially in Espírito Santo, which after 2010 stood out for presenting the lowest averages in the region, in a stable and sustained pattern. The FUs in the South region ( Figure 1D ), as observed in the Southeast region, presented increased averages at the beginning of the observation period and, with the exception of Paraná, which showed an increase throughout the 1990s, all other FUs showed a progressive decline, reaching values similar to those of the Southeast region in the final observation period. Figure 1E shows important fluctuations across the FUs in the Midwest region along the first three decades, trending toward linearity only over the last 10 years of observation. As seen separately in each region, the national trend pointed downward in the period ( Figure 1F ). After starting from intermediate values at the beginning of the series, small fluctuations occurred, especially in the 1990s, with a later trend toward linearity after the beginning of the 2000s.

Table 1 shows the ratio of the mortality rates between men and women across the five GRs at 5-year periods and in all three age groups. The rates in men were greater during almost all periods and age groups observed, reaching the highest proportions in the age group between 30–59 years in all GRs. The mortality rates among women were only higher (ratio <1) in the age group up to 29 years in brief periods in the North and Northeast regions, and in the age group above 60 years after 1995 in the South region ( Table 1 ).

**Table 1:** – Ratio between crude mortality rates in men and women in different age groups and by geographic region over 5-year periods

Age Group	Region/Period	1980-1984	1985-1989	1990-1994	1995-1999	2000-2004	2005-2009	2010-2014	2015-2018
0-29	North	1.0	0.9	1.2	1.0	1.2	1.3	1.3	1.8
	Northeast	0.9	1.0	1.0	1.1	1.1	1.4	1.4	1.5
	Southeast	1.1	1.2	1.3	1.3	1.2	1.6	1.5	1.8
	South	1.1	1.2	1.2	1.6	1.5	1.3	1.5	1.1
	Midwest	1.1	1.1	1.1	1.1	1.9	1.5	2.7	1.0
30-59	North	1.5	1.4	1.5	1.4	1.6	1.9	1.8	1.5
	Northeast	1.2	1.3	1.4	1.3	1.3	1.4	1.5	1.6
	Southeast	1.4	1.5	1.6	1.5	1.6	1.7	1.6	1.5
	South	1.4	1.5	1.5	1.4	1.4	1.5	1.4	1.2
	Midwest	1.2	1.5	1.6	1.6	1.8	1.9	1.7	1.7
60+	North	1.1	1.1	1.1	1.1	1.2	1.3	1.3	1.2
	Northeast	1.2	1.2	1.2	1.1	1.2	1.2	1.2	1.2
	Southeast	1.1	1.1	1.0	1.0	1.0	1.0	1.0	1.0
	South	1.1	1.0	1.0	0.9	0.9	0.9	0.9	0.9
	Midwest	1.1	1.1	1.0	1.0	1.1	1.1	1.1	1.2

The Pearson's correlation coefficient of the variation in mortality rates between 1990 and 2018 and the variation in MHDI between 1991 and 2010 for each FU was 0.73 (strong correlation), with p = 0.000. Figure 2A shows a scatter plot of the correlation across all FUs, while Figure 2B shows the correlation of mortality rates and MHDI in 2010, with a coefficient of 0.72. In line with Figure 1 , in relation to the moving averages over a longer period, all FUs showed reductions and, therefore, a negative variation in mortality rates comparing the years 1990 and 2018 ( Figure 2A , Y axis). In contrast, all FUs showed an increase and, therefore, a positive variation in the MHDI between 1991 and 2010 ( Figure 2A , X axis). As shown in Figure 2A , the FUs with the greatest reductions in mortality rates were those with the smallest increases in MHDI. In turn, those FUs with the smallest reductions in mortality rates were the ones that showed the largest increases in MHDI. Figure 2B shows the inverse relationship between the 2010 MHDI and the percentage changes in mortality rates. Table 2 shows the 2010 MHDI and the MHDI variations between 1991 and 2010 for each FU.

**Figure 2:** – *Shows a scatter plot. A) Correlation between the percentage variations of the MHDI between 1991 and 2010 and the mortality rates between 1990 and 2018, in each Federation Unit (Acronyms) in Brazil. B) Correlation between the absolute MHDI in 2010 and the percentage change in mortality rates between 1990 and 2018, in each Federation Unit (acronyms) in Brazil.*

**Table 2:** – Municipal Human Development Index (MHDI) by Federative Unit and variation between 1991 and 2010

Federative Unity	2010 MHDI	∆% 1991-2010
Rondônia	0.690	69.5
Acre	0.663	64.9
Amazonas	0.674	56.7
Roraima	0.707	54.0
Pará	0.646	56.4
Amapá	0.708	50.0
Tocantins	0.699	89.4
Maranhão	0.639	79.0
Piauí	0.646	78.5
Ceará	0.682	68.4
Rio Grande do Norte	0.684	59.8
Paraíba	0.658	72.3
Pernambuco	0.673	53.0
Alagoas	0.631	70.5
Sergipe	0.665	63.0
Bahia	0.660	71.0
Minas Gerais	0.731	52.9
Espírito Santo	0.740	46.5
Rio de Janeiro	0.761	32.8
São Paulo	0.783	35.5
Paraná	0.749	47.7
Santa Catarina	0.774	42.5
Rio Grande do Sul	0.746	37.6
Mato Grosso do Sul	0.729	49.4
Mato Grosso	0.725	61.5
Goiás	0.735	50.9
Distrito Federal	0.824	33.8

*∆% 1991-2010 = percentage change between 1991 and 2010.*

## Discussion

Worldwide, HF affects approximately 26 million people. This number tends to increase with population aging, increased prevalence of cardiovascular risk factors, survival of patients to acute coronary events, and therapeutic improvements in HF.^14^ Estimates in the United States project more than 8 million people with HF by 2030, with the numbers increasing due to population aging.^15^

Mortality rates due to HF have decreased in Brazil over the 29 years analyzed in the present study, showing a trend toward a progressive reduction after 2008 and reaching similar levels across GRs and FUs by the end of 2018 ( Figure 1 ). This trend was similar to that observed in a study with 5,823 patients followed up for 1 year across different regions worldwide, which showed a proportional mortality of 9% in South America. The authors observed high mortality rates in Africa (34%) and India (23%), followed by intermediate rates in Southeast Asia (15%) and lower rates in China and the Middle East (7%), which persisted despite adjustment for multiple clinical variables, medication therapy, and socioeconomic factors. The authors hypothesized that the quality, access, and infrastructure of the health services, as well as genetic and environmental factors, could be involved in this complex phenomenon.^16^

Another important aspect is that the average age of the patients with HF was a decade younger in low- and middle-income countries compared with high-income countries,^17^ which may be related to the delay in diagnosis and treatment that could lead to a worse prognosis for less favored patients, adding to the low life expectancy in these countries.^14 , 18^ In a cohort of 4 million individuals from the Clinical Practice Research Datalink (CPRD) and representative of the UK population, socioeconomically disadvantaged individuals were more likely to develop HF than wealthy individuals (rate ratio incidence 1.61, 95% CI 1.58–1.64), and did so earlier in life (adjusted difference -3.51 years, 95% CI 3.77-3.25) and with more comorbidities despite the younger age. They also observed an increase in the socioeconomic gradient in the age at the first HF presentation between 2002 and 2014.^19^

Mortality rates due to HF in men were higher during almost all periods and age groups observed, except in the age group above 60 years after 1995 in the South region ( Table 1 ), probably related to the ischemic etiology of HF, except at more advanced ages, which may be associated with longer longevity in women, as observed in a meta-analysis of about 240,000 patients with acute and chronic HF.^17^ Another study of 88,416 patients also from the UK database CPRD observed that the risks of adverse outcomes were greater in individuals who are older, men, with socioeconomic limitations, and in those whose HF diagnosis was established during hospitalization. They also observed worse outcomes in women over the past two decades. The authors concluded that these disparities probably reflect the growing burden of non-CVDs in patients with HF, which will require changes in the contemporary approach and, in turn, will need to incorporate management and improvement in socioeconomic status.^20^

Previous studies have shown that patients have HF at a younger age in countries with lower compared with higher HDI,^17^ and that economic limitations are associated with a higher incidence of HF at a national level.^21 , 22^ A study with more than 17,100 patients with HF and reduced left ventricular ejection fraction from a Universal Health System has reported that low income was associated with a higher risk of death from all causes, readmission within 12 months from the HF diagnosis, longer hospital stay, and higher hospital mortality rate.^23^

An inverse trend was observed between the variation in mortality rates in the FUs between 1990 and 2018 and the variation of the respective MHDIs between 1991 and 2010. Thus, although the FUs that presented the greatest reductions in mortality rates showed the smallest increases in MHDI ( Figure 2A - RJ, DF, SP, RS, SC, ES), they all reached MHDI equal to or greater than 0.7 in 2010 ( Table 2 ). In contrast, none of the FUs with the largest increments in MHDI ( Figure 2A - TO, MA, PI, PB, AL, BA) had an MHDI greater than 0.7 in 2010 ( Figure 2B ). This fact suggests that in relation to HF mortality, more important than the degree of MHDI increase is the final MHDI level. A study evaluating 1,802 UK patients with HF and reduced left ventricular ejection fraction using an Index of Multiple Deprivation found that all-cause mortality and mortality due to noncardiac causes adjusted for age were associated with a high risk of socioeconomic limitation, but not with mortality from cardiovascular causes. This excess risk was attributed to excess noncardiac mortality and hospitalizations and cannot be associated with the lack of medication for evidence-based HF. The authors suggest that socioeconomic interventions need to be implemented to reduce the personal risks and economic burden of the disease in patients with HF and low socioeconomic status.^24^

In the present study, we did not evaluate multiple causes of death, only the underlying causes of death selected from the information recorded in the death certificates. This fact becomes a limitation because the codes related to HF are generally discarded after the application of the rules for the selection of underlying causes by the World Health Organization,^25^ which can lead to an undersized estimate of deaths due to HF. However, as these are rules of global applicability, it is believed that there is no loss when comparing deaths between different countries and/or regions.

Another point to be highlighted is that since this is a study that directly evaluates the underlying cause of death, the quality of the information depends on the proper completion of the death certificate. Errors in completion and incompleteness of the certificates caused by lack of knowledge of the declarant^26^ represent potential problems that can interfere with official statistics. However, because they are systemic in nature, possible errors could affect all the causes of death, influencing not only those deaths due to HF.

Despite including data related to income, education level, and life expectancy, the HDI represents only a partial view of the socioeconomic status of a given country or region, thus not assessing phenomena such as inequality or quality of life and their influences on HF mortality. However, since this index is available worldwide, it allows for comparing, with proper dimensioning, different populations.

In addition to representing an enormous burden for society, HF is the main cause of hospitalization in western countries.^27^ The increasing prevalence of HF, especially in younger individuals, and the inefficient public spending on health care in developing countries, which already have important social inequalities, will require a reconsideration of the economic impact of HF, especially in a country with continental dimensions like Brazil.

Few data are known about the epidemiology of HF, especially in middle-income countries like Brazil, where the prevalence of HF is believed to be increasing, and an increasing association has been reported with ischemic heart disease, rheumatic disease, Chagas disease, and hypertension, among others.^28^ The reduction in HF mortality may be a consequence of advances in the treatment of ischemic heart disease, but it must also be related to the progression in HF treatment itself, especially after the introduction of neurohumoral blocker therapy.^4^

Efforts must be made to expand access to health care and toward more effective control of cardiovascular risk factors, dyslipidemia, obesity, physical inactivity, and diabetes, as well as social determinants that contribute to both mortality from HDI and HF. It is within this context that the scope of the Family Health Program can play an important role, which in addition to attributing the model of the care to primary care, increases the coverage of the National Health System, reducing the proportion of unattended deaths, improving the quality of vital information in Brazil, and decreasing hospitalizations due to chronic diseases such as HF.^29^ Future studies must be carried out to analyze the association of the installed capacity of health care resources, and the multiple causes represented by risk factors as contributors to the complex process of death to improve the guidance of public health policies regarding HF in Brazil.

## Conclusion

This study evaluating HF mortality in Brazil over 39 years at each FU of the GRs demonstrated that, despite variations, all FUs showed a reduction in mortality rates, especially in the last 10 years of observation. A predominance of deaths in men was observed in the age group of 30–59 years. There was a trend toward an inverse relationship between the percentage of increase in MHDI and reduction in mortality rates, the latter potentially related to the absolute level of MHDI achieved in 2010. These findings could, at least in part, be justified by improved access to the health system in HF treatment and socioeconomic conditions of the population over almost four decades.

## * Supplemental Materials

For additional information, please click here:
